# Supplementary material for: Performance of a Point of Care Test for Detecting IgM and IgG Antibodies Against SARS-CoV-2 and Seroprevalence in Blood Donors and Health Care Workers in Panama
Source: Front Med (Lausanne). 2021 Mar 2;8:616106. doi: 10.3389/fmed.2021.616106 (PMC7968482; doi:10.3389/fmed.2021.616106)
Supplement: Supplementary Figure 1 — Representative picture of CAST IgM and IgG antibody test results. (A) Only IgM; (B) Only IgG; (C) Both IgM and IgG; (D) No IgM or IgG. [file Data_Sheet_1.docx]

**Supplementary table. Symptoms among COVID-19 patients.**

| Symptoms | COVID19 participant  # (%) |
| --- | --- |
|  | **Total**  **(N=96)** |
| Cough | 73 (76.0%) |
| Dyspnea | 68 (70.8%) |
| Fever | **64 (66.7%)** |
| Diarrhea | 56 (58.9%) |
| Headache | 52 (54.2%) |
| Fatigue | 50 (49.0%) |
| Chills | 45 (58.4%) |
| Muscular pain | 39 (40.6%) |
| Chest pain | 39 (40.6%) |
| Sore throat | 37 (38.5%) |
| Nausea or vomit | 34 (35.4%) |
| Rhinorrhea | 29 (30.2%) |
| Abdominal pain | 29 (30.2%) |
| Wheezing | 21 (21.9%) |
| Other breathing symptoms | 8 (8.4%) |

Supplementary Figure 1:


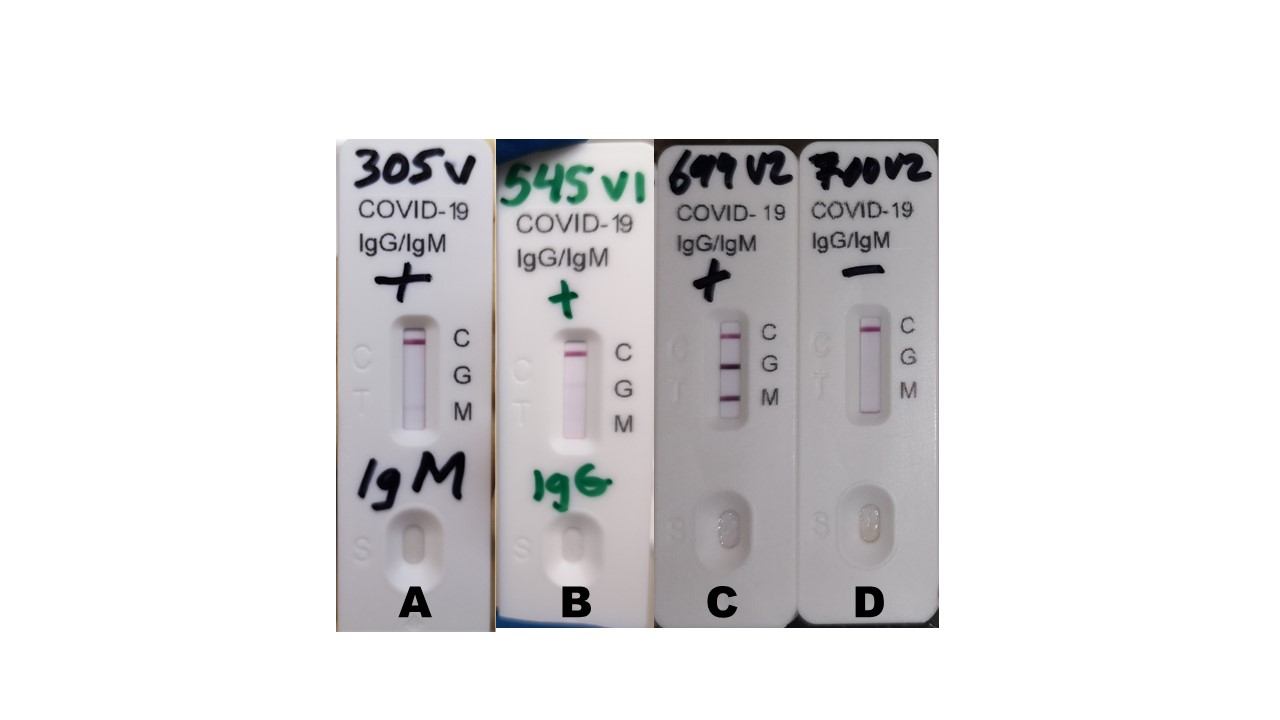


**Supplementary figure 1:** Representative picture of CAST IgM and IgG antibody test results. Panel A: Only IgM; Panel B: Only IgG; Panel C: Both IgM and IgG; Panel D: No IgM or IgG.
